# A novel film spray containing curcumin inhibits SARS-CoV-2 and influenza virus infection and enhances mucosal immunity

**DOI:** 10.1186/s12985-023-02282-x

**Published:** 2024-01-23

**Authors:** Wipawee Nittayananta, Hatairat Lerdsamran, Nopporn Chutiwitoonchai, Aornrutai Promsong, Teerapol Srichana, Kesinee Netsomboon, Jarunee Prasertsopon, Jaruta Kerdto

**Affiliations:** 1 Faculty of Dentistry, Thammasat University, Pathum Thani, Thailand; 2 Center for Research Innovation and Biomedical Informatics, Faculty of Medical Technology, Mahidol University, Nakhon Pathom, Thailand; 3 National Center for Genetic Engineering and Biotechnology, National Science and Technology Development Agency, Pathum Thani, Thailand; 4 Faculty of Medicine, Princess of Naradhiwas University, Narathiwat, Thailand; 5 Drug Delivery System Excellence Center, Faculty of Pharmaceutical Sciences, Prince of Songkla University, Hat Yai, Songkhla Thailand; 6 Department of Pharmaceutical Technology, Faculty of Pharmaceutical Sciences, Prince of Songkla University, Hat Yai, Songkhla Thailand; 7 Faculty of Pharmacy, Thammasat University, Pathum Thani, Thailand; 8 Thammasat Hospital, Thammasat University, Pathum Thani, Thailand

**Keywords:** COVID-19, Curcumin, Influenza, Mucosal immunity, SARS-CoV-2

## Abstract

**Background:**

Infection by severe acute respiratory syndrome coronavirus-2 (SARS-CoV-2) and influenza virus is still a major worldwide health concern. Plants are a good source of bioactive compounds to be used as preventive measures for both inhibiting the virus binding and enhancing mucosal innate immunity. Curcumin has been shown to possess antiviral activity and modulate innate immunity. Therefore, the purpose of this study was to develop an oro-nasal film spray containing curcumin and determine its antiviral activity against SARS-CoV-2 and influenza virus infection, as well as its effects on mucosal innate immunity and inflammatory cytokines in vitro.

**Methods:**

The antiviral activity of the film spray against SARS-CoV-2, influenza A/H1N1, A/H3N2, and influenza B was assessed in vitro by plaque reduction assay. Cytotoxicity of the film spray to oral keratinocytes and nasal epithelial cells was assessed by MTT assay, and cytotoxicity to Vero and MDCK cells was assessed by an MTS-based cytotoxicity assay. Oral and nasal innate immune markers in response to the film spray were determined by ELISA and by a commercial Milliplex Map Kit, respectively.

**Results:**

Our data show that the film spray containing curcumin can inhibit both SARS-CoV-2 and influenza virus infections while maintaining cell viability. Results obtained among 4 viruses revealed that curcumin film spray demonstrated the highest inhibitory activity against SARS-CoV-2 with the lowest EC_50_ of 3.15 µg/ml and the highest SI value of 4.62, followed by influenza B (EC_50_ = 6.32 µg/ml, SI = 2.04), influenza A/H1N1 (EC_50_ = 7.24 µg/ml, SI = 1.78), and influenza A/H3N2 (EC_50_ > 12.5 µg/ml, SI < 1.03), respectively. Antimicrobial peptides LL-37 and HD-5, IL-6 and TNF-α produced by oral keratinocytes were significantly induced by the film spray, while hBD2 was significantly reduced.

**Conclusion:**

Film spray containing curcumin possesses multiple actions against SARS-CoV-2 infection by inhibiting ACE-2 binding in target cells and enhancing mucosal innate immunity. The film spray can also inhibit influenza virus infection. Therefore, the curcumin film spray may be effective in preventing the viral infection of both SARS-CoV-2 and influenza.

## Background

Coronavirus disease 2019 (COVID-19) has rapidly spread throughout the world since 2019 [1]. It is an infectious disease caused by severe acute respiratory syndrome coronavirus-2 (SARS-CoV-2), an enveloped RNA virus with a genome encoding the spike (S) protein, envelope protein, membrane protein, nucleoprotein, and various non-structural proteins. The illness commonly known as “flu” or “the flu” is a seasonal respiratory infection caused by influenza virus types A, B, and C; however, type A is the most virulent [2]. Both COVID-19 and flu can affect all age groups and are associated with high mortality, especially in elderly patients and patients with immunocompromised conditions [2]. Although efforts have been made towards preventive measures, infections by SARS-CoV-2 and influenza virus, especially type A (IVA), are still providing challenges for medical and public health systems.

The pathogenesis of SARS-CoV-2 and IVA infection may involve several stages, including the suppression of host antiviral and innate immune responses [3, 4]. The viral replication-induced infected cell secretes various chemokines or cytokines, such as interleukins (IL-1, IL-6, IL-8, IL-12), interferons (IFNs), tumor necrosis factor-α (TNF-α), and colony-stimulating factors, causing acute inflammation described as the “cytokine storm symptom” [5, 6]. Both SARS-CoV-2 and influenza virus are potent stimulators of pro-inflammatory cytokine production which correlates with lung injury. Thus, a major goal in the treatment of these infectious diseases should be to reduce the inflammatory effects of the viruses.

Currently, treatment for COVID-19 and flu are based on signs and symptoms and may include antiviral agents and steroids [7], which can cause adverse side effects [8]. Although co-infection of SARS-CoV-2 and IVA is rare, they share similar signs and symptoms and individuals with both infections may be at higher risk of poor health outcomes [9, 10]. Because presently there is no conclusive information on the effectiveness of approved drugs for COVID-19 and flu, efforts should be made to identify natural compounds with potent antiviral activity against both viruses.

Plants are the major source for new drug discoveries to combat infectious disease [11]. Among these, curcumin is a promising candidate as it shows antiviral activity against both SARS-CoV-2 and IVA [12, 13]. A recent study reported that curcumin inhibited SARS-CoV-2 D614G strain by pre-infection treatment of Vero E6 cells [12]. This effect has also been observed with other enveloped viruses including IVA [13, 14]. Previous studies have reported that curcumin prevents SARS-CoV-2 entry by direct interaction with cell factors or viral proteins [15, 16]. Therefore, it is encouraging to investigate curcumin as a potential drug candidate to suppress the virus and its application in preventing COVID-19 and flu.

Mucosal innate immunity plays a crucial role in the development of infections. Antimicrobial peptides produced by epithelial cells lining oro-nasal cavity, including human cathelicidin LL-37 and alpha- and beta-defensins, are part of the mucosal innate immunity system. These antimicrobial peptides have broad-spectrum antimicrobial activities and participate in mucosal innate immunity against SARS-CoV-2 and IVA [17–19]. Because human-to-human transmission through droplets is the main mode of both SARS-CoV-2 and influenza virus transmissions, the oral and nasal cavities represent the major routes and reservoirs for the viruses [20]. Thus, inhibiting viral infection at the port of entry and enhancing oral and nasal innate immunity may be a novel strategy for prevention of COVID-19 and flu infections.

Recently, blocking viral entry with phytochemical compounds to prevent infection has been intensively studied. Curcumin has been documented to possess antiviral activity and modulate innate immunity [1]. Therefore, it may be used as a preventive measure for both inhibiting the virus binding and enhancing mucosal innate immunity. The purpose of this study was to develop a novel oro-nasal film spray containing curcumin and determine its antiviral activity against SARS-CoV-2 and influenza virus infection, as well as its effects on mucosal innate immunity and inflammatory cytokines in vitro.

## Materials and methods

### Preparation of curcumin

Curcumin was a pure compound purchased from Sigma-Aldrich (Sigma-Aldrich, Saint Louis, MO, USA). The curcumin powder was dissolved in ethanol to form a stock solution of 500 µg/ml, and the final dilution was used as 10 µg/ml or dissolved in dimethyl sulfoxide (DMSO) to form a stock solution of 20 mg/ml for the pseudotyped virus entry assay.

### Preparation of a film spray containing curcumin

Formulation of a film spray containing the active ingredient curcumin (10 µg/ml) was prepared by modifying the method used in a previously published paper [21]. Briefly, ethanol (15% w/v), polyethylene glycol (20% w/v) and tween 80 (2% w/v) were mixed. Then, xylitol (10% w/v) and menthol (0.3% w/v) were added to the mixture. Polyvinylpyrrolidone, EDTA, and sodium benzoate were separately hydrated and added to the previous mixture to produce concentrations of 5, 0.1 and 0.1% w/v of the formulation, respectively. Afterwards, curcumin (0.001% w/v) was added to the mixture. Deionized water was used for final volume adjustment. The physical properties of the formulation were recorded before and after the stability study (Freeze-thaw 5 cycles).

### Cell lines and pseudotyped virus

Human embryonic kidney cells (HEK293T/17 cells) stably expressing human ACE2 (HEK293-hACE2) were generated by lentivirus transduction, following the methods previously described [22, 23]. The cells were cultured in Dulbecco’s Modified Eagle Medium (DMEM)/High glucose (Hyclone Laboratories, Logan, UT, USA) supplemented with 10% (v/v) fetal bovine serum and antibiotics. The pseudotyped virus harboring Wuhan SARS-CoV-2 spike protein (SARS-CoV-2 spike pseudotyped virus) was generated as mentioned in a previous study [23, 24].

### SARS-CoV-2 and influenza viruses

To determine the antiviral activity of curcumin film spray against SARS-CoV-2 and influenza viruses, SARS-CoV-2 Variant 20I (Alpha, V1) or B.1.1.7 was isolated from a clinical sample from a COVID-19 patient in Thailand and propagated in Vero cells maintained in Eagle’s Minimum Essential Medium (EMEM) (Gibco, NY, USA) supplemented with L-glutamine, antibiotic-antimycotic, and 2% FBS. In contrast, three strains of influenza viruses including influenza A/H1N1 (A/Puerto Rico/8/1934, ATCC VR-95), influenza A/H3N2 (A/Aichi/2/1968 H3N2, ATCC VR-547), and influenza B (B/TH/MUMT-1/2015) were propagated in MDCK cells and maintained in EMEM supplemented with L-glutamine, antibiotic-antimycotic, and 2 µg/ml trypsin-tosyl phenylalanyl chloromethyl ketone (Trypsin-TCPK) (Sigma Aldrich, St. Louise, MO, USA).

All tested viruses were cultured under humidified (37 °C, with 5% CO_2_) conditions in a Biosafety Level 3 (BSL-3) laboratory (for SARS-CoV-2) and in a BSL-2 laboratory (for influenza viruses). The aliquots of the stock viruses were stored at below − 70 °C until used in the assays.

### SARS-CoV-2 spike pseudotyped virus infection assay

The effects of the curcumin compound and the film spray formulation containing the compound with spike-ACE2-mediated SARS-CoV-2 infection were evaluated using the surrogate assay system with SARS-CoV-2 spike pseudotyped virus and HEK293-hACE2 cells expressing human transmembrane protease serine-2 (TMPRSS-2), as previously described [23, 24]. Briefly, the virus was pre-incubated with each sample in an opaque 96-well white microplate (PerkinElmer, Waltham, MA, USA) at 37 °C for 1 h and then introduced into the host cells. The virus treated with DMSO was used as a control to represent 100% viral infectivity. After 48 h, viral infectivity was determined by measuring the activity of the viral reporter, luciferase, using the Bright-Glo Luciferase Assay System (Promega) and Synergy HTX Multi-Mode Microplate Reader (BioTek, Winooski, VT, USA).

### Plaque reduction assay

Plaque reduction assay is the current standard phenotypic method for in vitro antiviral susceptibility testing. For the antiviral activity screening, curcumin film spray was included in the reaction throughout the steps of virus replication including pre-treatment, co-treatment, and post-treatment. Briefly, confluent cell monolayers were prepared in 12 well plates. Cells were pre-treated with various concentrations of the film spray samples at 37 °C, 5% CO_2_ for 1 h. After that, the test virus at a concentration of 50 PFU/well was added into each well and incubated at 37 °C, 5% CO_2_ for 1 h. Then, the mixture was removed, and infected cells were overlaid with 1.2% Avicel in the presence of the samples as post-treatment and further incubated for 72 h. After fixing and staining with 1% crystal violet, the viral plaques were counted and percentages of inhibition at each sample concentration were calculated as compared with the untreated virus controls. Virus control (without the samples) and cell control (without samples and virus) were run in parallel. The 50% effective concentration (EC_50_) of the film spray was determined from the dose-response curve using GraphPad Prism 10.0 Software. The concentration of film spray samples that reduces the number of viral plaques by 50% in comparison to untreated virus control is EC_50_. The selectivity index (SI), the ratio between cytotoxicity and antiviral activity of the test sample, was calculated as the CC_50_/EC_50_ ratio.

### Cell culture conditions

#### Vero cells and MDCK cells

The Vero cells (African green monkey kidney cells) from American Type Culture Collection (ATCC), CCL-81) and MDCK (Madin-Darby Canine Kidney) cells (from ATCC,CCL-34) were grown in EMEM (Gibco, NY, USA) supplemented with 10% heat-inactivated fetal bovine serum (FBS) (Gibco, NY, USA), 5% sodium bicarbonate, L-glutamine (Hyclone, South Logan, UT, USA), penicillin-streptomycin (Gibco, NY, USA), and amphotericin B (Gibco, NY, USA) in humidified conditions (37 °C, with 5% CO_2_).

#### Oral keratinocyte cell line

The oral keratinocyte (OKC) cell line was provided by the Faculty of Dentistry, Chulalongkorn University, Bangkok, Thailand. The cells were cultured as previously described [25]. Briefly, the cell line was grown in Defined Keratinocyte Serum Free Media (Gibco, NY, USA), supplemented with Keratinocyte Growth factors (Gibco, NY, USA) and 100 U/ml antibiotic-antimycotic (Gibco, NY, USA). The cells were incubated at 37ºC in a 5% CO_2_ atmosphere until reaching 80% confluence, trypsinized with 0.25% trypsin-EDTA (Gibco, NY, USA) and the enzyme activity was inactivated with 10% FBS (Gibco, NY, USA) in 1x PBS. Cell viability was examined using 0.4% trypan blue (Gibco, NY, USA) staining with a light microscope.

#### Human nasal septum epithelial cell line

The human nasal septum epithelial cell line (RPMI 2650, ATCC: CCL-30, MD, USA) was cultured in EMEM (Gibco, NY, USA) supplemented with 10% FBS (Gibco, NY, USA), and 100 U/ml of penicillin/streptomycin (Gibco, NY, USA). Cells were incubated at 37ºC in a 5% CO_2_ incubator and the media was changed every 2 days. They were harvested by gentle rocking, followed by the addition of fresh culture medium to create a new single cell suspension for further incubation.

### Cytotoxicity assay

The HEK293-hACE2 cells used in the pseudotyped virus entry assay were tested for cytotoxicity of the curcumin film spray samples. Briefly, the cells were seeded (4 × 10^5^ cells/ml) in a 96-well plate and incubated at 37ºC in 5% CO_2_ for 24 h. The cells were treated with samples at different concentrations for 48 h. Cells without a sample (DMSO-treated) served as a negative control (100% cell viability). Viability of the treated cells was measured by the Cell Counting Kit-8 (CCK-8) reagent (Dojindo, Rockville, MD, USA) using Synergy HTX Multi-Mode Microplate Reader (BioTek, Winooski, VT, USA).

Both Vero and MDCK cells were tested for cytotoxicity of the curcumin film spray samples with an MTS-based cytotoxicity assay using CellTiter 96®AQueous One Solution Cell Proliferation Assay kit (Promega, WI, USA). Briefly, monolayer cells in 96-well plates were treated with serial 2-fold dilutions of the film spray starting from 100 to 0.1 µg/ml in quadruplicate wells. Cells without treatment served as mock control. Cells were incubated at 37 °C with 5% CO_2_ for 72 h, then MTS solution was added into each well and incubated at 37 °C with 5% CO_2_ for 3 h. Cell viability after treatment was determined by measuring the optical density (OD) of the formazan product in each reaction well at a wavelength of 490 nm. The percentage of viable cells from each concentration of drug treatment was calculated by comparing it with the untreated control cells and determined for the 50% cytotoxic concentration (CC_50_) from the dose-response curve using GraphPad Prism 10.0 Software.

Oral and nasal keratinocytes (1 × 10^5^ cells/well) were tested for cytotoxicity of the curcumin film spray samples. The cells were seeded in a 96-well plate and incubated at 37ºC in 5% CO_2_ for 24 h. After incubation, 1.25, 2.5, 5, 10, 20 µg/ml of the film spray were added into the culture plates of the cells. Cells without sample served as a negative control. After 24 h incubation, methylthiazol tetrazolium (MTT) assay was performed to evaluate cell activity, as previously described (Promsong et al., 2015). In brief, the cells were treated with 80 µl of fresh media along with 20 µl of MTT solution and incubated at 37ºC under 5% CO_2_ for 4 h. Afterwards, media containing MTT were removed and 100 µl of DMSO were added. The absorbance was determined by a microplate reader (Biohit 830, Helsinki, Finland) at a wavelength of 570 nm. The percentage of cell proliferation was calculated and compared to a negative control.

### Assessment of antimicrobial peptides

Effects of the film spray containing curcumin on mucosal innate immunity were determined by measuring levels of antimicrobial peptides, including LL-37, HD-5, and human β defensin 2 (hBD-2) in supernatant of the oral and nasal keratinocyte cell culture by ELISA kits (MyBioSource, CA, USA). Experiments were performed in duplicate, according to the manufacturer’s instructions. These kits are an antibody sandwich enzyme immunoassay. Each 96-well microplate was pre-coated with an antibody specific to the target peptide. Standards or samples were added to the plate wells and combined with a biotin-conjugated specific antibody. Then avidin-horseradish peroxidase conjugate was added to each plate well and incubated. After free components were washed away, substrate solution was added to each well. Enzyme-substrate reaction presented blue color development, which was terminated by addition of stop solution. After which, the color turned yellow. Finally, the color intensity was measured at 450 nm for test wavelength and 630 nm for reference wavelength (CLARIOstar^Plus^, BMG LABTECH GmbH, Germany). The quantity of target analyte in the sample was positively correlated by comparison with the standard curve.

### Determination of the effects of the film spray on inflammatory cytokines

Cytokine levels were determined using a multiplexed bead immunoassay. The cell supernatant sample was analyzed for IL-1β, IL-6, IL-10, TNF-α, and IFN-γ using a commercially available high-sensitivity human cytokine panel (Milliplex Map Kit; Millipore, Billerica, MA, USA) within a Luminex analyzer. Briefly, 25 µl of standards, quality controls and samples are added to the plate in duplicate. This was followed by 25 µl of magnetic beads. The plate was sealed, covered with foil, and incubated overnight on a plate shaker at 4 °C. The plate was washed three times and 25 µl of detection antibody was added to each well. After incubating the plate at room temperature (RT) for 1 h, 25 µl of streptavidin-phycoerythrin (PE) was added, per well. The plate was resealed and incubated for an additional 30 min at RT. The plate underwent a final series of washes before 150 µl of drive fluid was added. The concentrations of markers were measured on a Luminex® MAGPIX® instrument with Belysa® Immunoassay Curve Fitting Software (Millipore, Billerica, MA, USA). Quality control values for each marker were consistently within the range indicated by the manufacturer.

### Statistical analysis

The results from SARS-CoV-2 spike pseudotyped virus infection assay and ELISA of antimicrobial peptides were recorded as mean ± SD of singlicate or duplicate cultures. The data were analyzed using Microsoft Excel and GraphPad Prism Software 9.5.0 (GraphPad Software, California, USA) and t-test to compare between the groups, respectively.

## Results

### Physical properties of the formulation

The most common delivery method for treating oral and nasal infection is via oromucosal/nasal film spray delivery. As curcumin has very poor water solubility, the ethanol and polyethylene glycol were used as cosolvents in the formulation system, and polyvinylpyrrolidone was used as mucoadhesive agent. EDTA was the chelating agent and sodium benzoate was used as a preservative. The flavoring agents were xylitol and menthol. The curcumin film spray was translucent yellow solution. There were no changes of formulation before and after freeze thaw, indicating that the formulation is stable (Table 1). The pH was 6.38, mildly acidic, and did not change significantly after freeze thaw to pH 6.20. The droplet sizes of the spray were 190–210 μm before and after freeze thawing and were suitable for spray droplets, either in the oral or the nasal cavities. The osmolarity was observed at 295–302 mOs/L, which is close to isotonic solution (300 Os/L) indicating that is unlikely to irritate oral or nasal epithelium. The viscosity is 50 cP at shear rate 50 s^− 1^. It is a shear-thinning system. Overall, there were no properties changes after freeze thawing, indicating that the formulation is stable.

**Table 1:** Physical properties of curcumin film spray (mean ± SD, n = 3)

Test	Initial	After freeze thaw
Appearance	Translucent yellow	Translucent yellow
pH	6.38 ± 0.02	6.20 ± 0.04
Droplet size (µm)	191 ± 4	211 ± 5
Osmolarity (mOs/L)	295 ± 2	302 ± 2
Viscosity (cP)	50 ± 2	52 ± 2

### Effects of curcumin compound on cell viability and viral infectivity

**Fig. 1:**
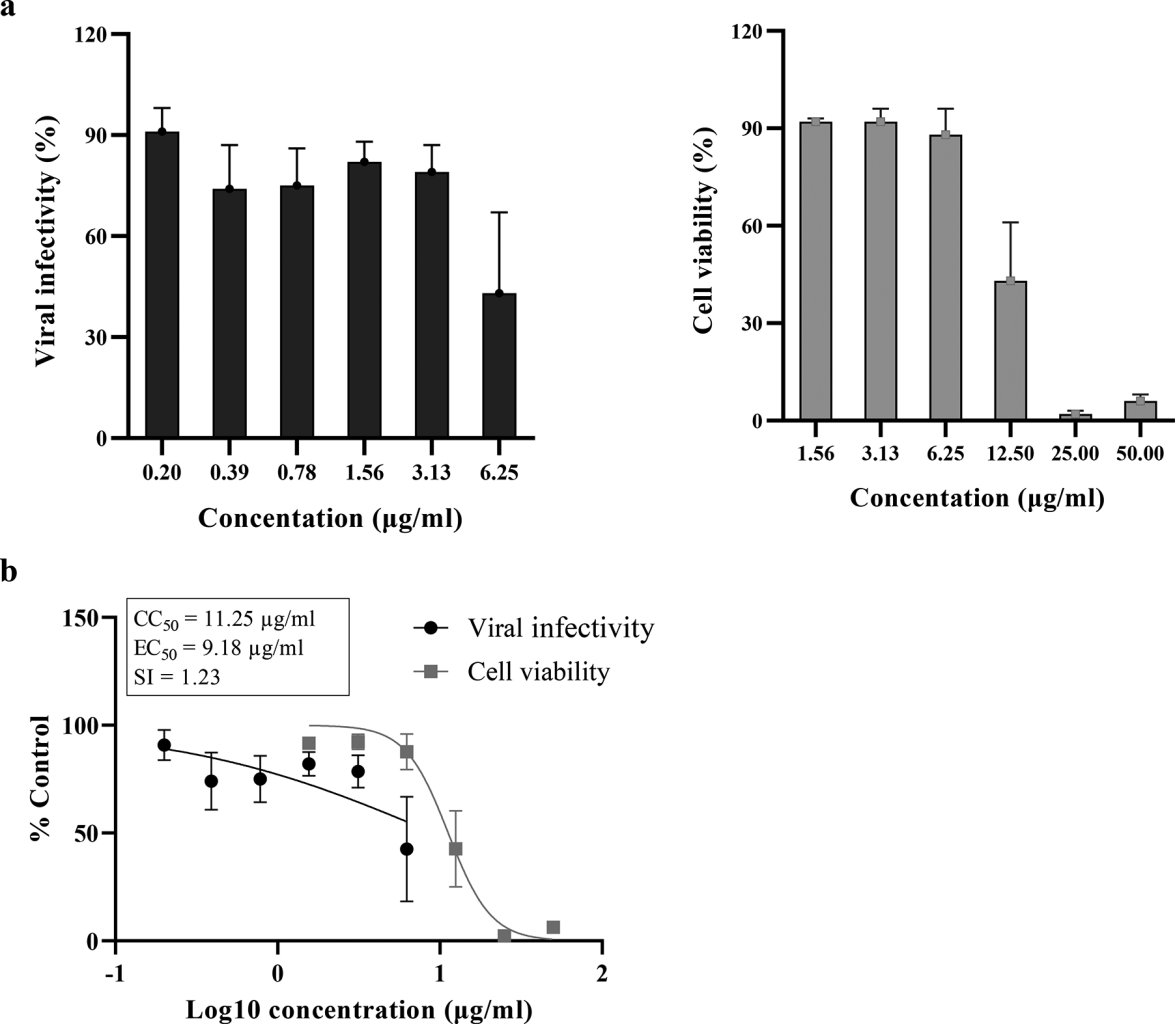
Infectivity and cytotoxicity of the curcumin compound at various concentrations. (**a**) The SARS-CoV-2 spike pseudotyped virus was treated with the compound at 37 °C for 1 h and infected into HEK293-hACE2 cells expressing TMPRSS-2. The virus treated with DMSO served as the control. After 48 h of incubation, viral infectivity was determined by measuring the activity of the luciferase reporter using the Bright-Glo Luciferase Assay System. The cytotoxicity of the compound was determined by the viability of treated cells without infection using the CCK-8 assay. The relative mean values ± SD (%DMSO) from 3–4 independent experiments (each performed in duplicate) are shown. (**b**) Data from (**a**) were fitted into dose-response curves using GraphPad Prism software, and the estimated CC_50_, EC_50_, and selectivity index are shown

The cytotoxicity of the curcumin compound on HEK293-hACE2 cells was determined by the CCK-8 assay. The effect of the compound on spike-ACE2 mediated SARS-CoV-2 infection was evaluated using the surrogate assay system with SARS-CoV-2 spike pseudotyped virus and HEK293-hACE-2 cells expressing human TMPRSS-2. The results showed that curcumin at 6.25 µg/ml can inhibit the pseudovirus while maintaining cell viability (Fig. 1a). Additionally, the compound inhibited the pseudovirus with a half maximal effective concentration (EC_50_) value of 9.18 µg/ml and a half maximal cytotoxic concentration (CC_50_) value of 11.25 µg/ml, resulting in a selectivity index of 1.23 (Fig. 1b).

### Effects of curcumin film spray on cell viability and viral infectivity

**Fig. 2:**
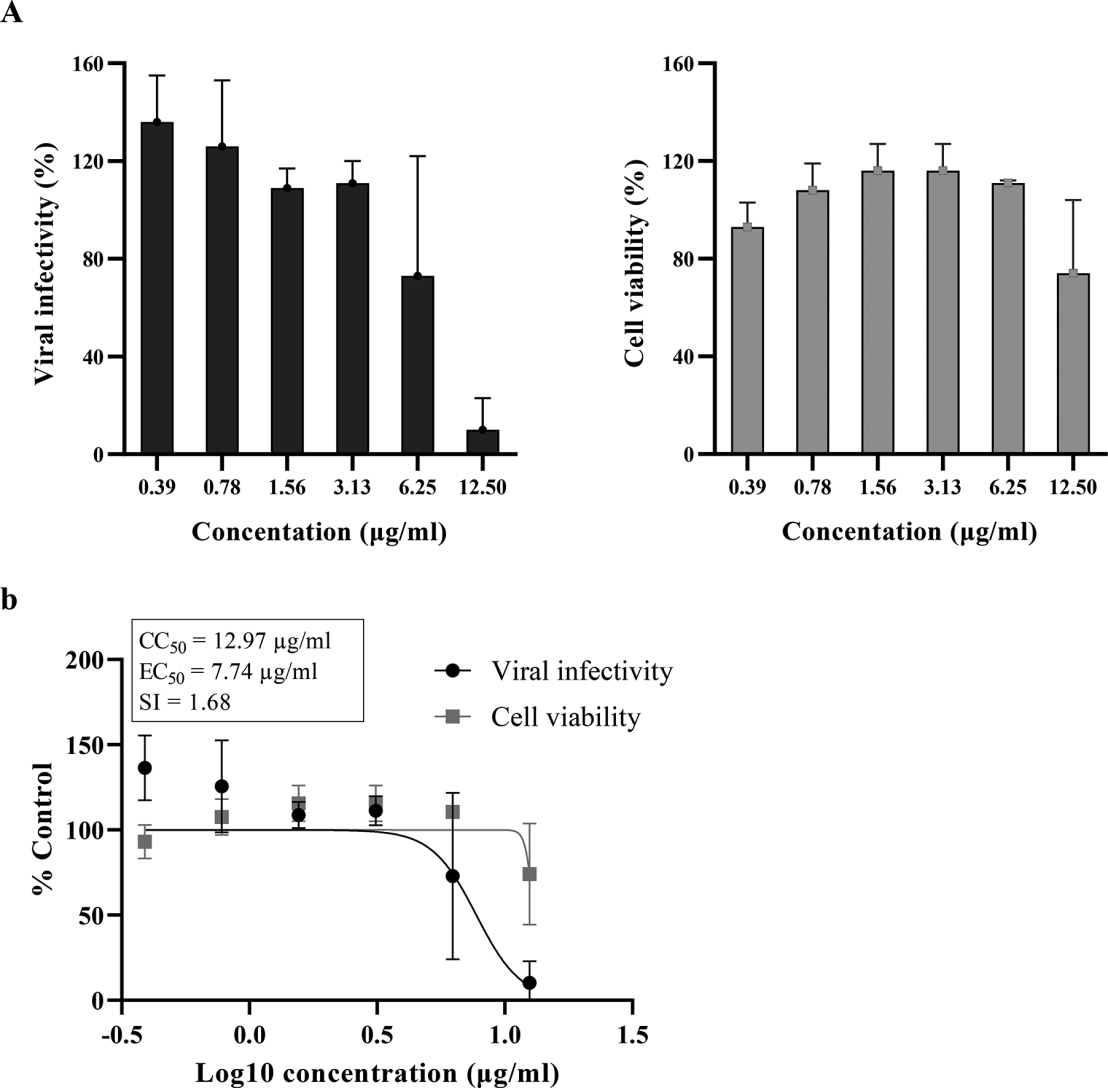
Infectivity and cytotoxicity of the curcumin film spray. (**a**) The SARS-CoV-2 spike pseudotyped virus was treated with the sample at 37 °C for 1 h and infected into HEK293-hACE2 cells expressing TMPRSS-2. The virus treated with the solvent (base) served as the control. After 48 h of incubation, viral infectivity was determined by measuring the activity of the luciferase reporter using the Bright-Glo Luciferase Assay System. The cytotoxicity of the sample was determined by the viability of treated cells without infection using the CCK-8 assay. The relative mean values ± SD (%DMSO) from 2 independent experiments (each performed in duplicate) are shown. (**b**) Data from (**a**) were fitted into dose-response curves using GraphPad Prism software, and the estimated CC_50_, EC_50_, and selectivity index are shown

The effect of the film spray formulation on spike-ACE2 mediated SARS-CoV-2 infection was evaluated using the surrogate assay system with SARS-CoV-2 spike pseudotyped virus and HEK293-hACE-2 cells expressing human TMPRSS-2, as mentioned earlier. The results showed that the spray, at a concentration of 10 µg/ml, can inhibit the pseudovirus while maintaining cell viability (Fig. 2). The estimated EC_50_ and CC_50_ values of the spray were 7.74 µg/ml and 12.97 µg/ml, respectively, resulting in a selectivity index of 1.68.

**Fig. 3:**
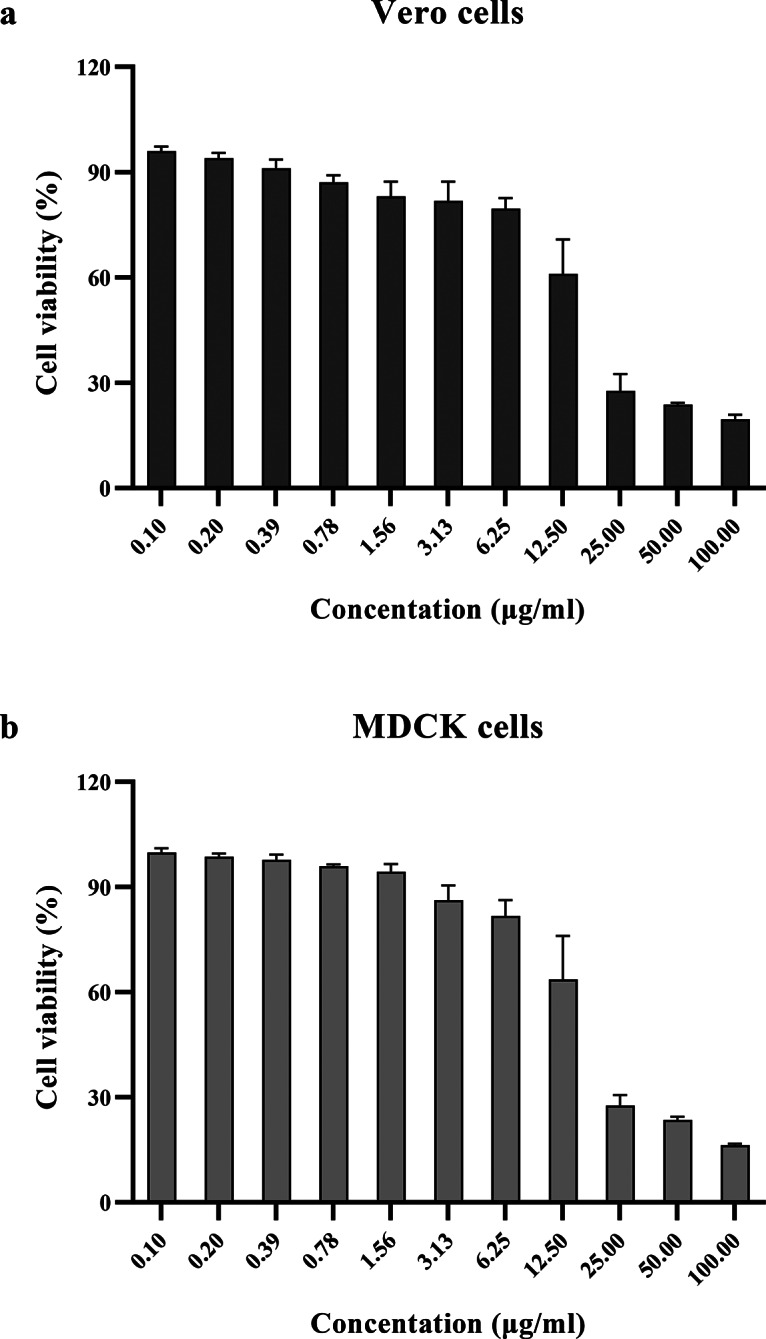
Cytotoxicity of curcumin film spray to Vero and MDCK cells. Vero cells (**a**) or MDCK cells (**b**) were treated with varying concentrations of the curcumin film spray for 72 h. Subsequently, cytotoxicity was determined by measuring viability of the treated cells using the CellTiter 96AQueous One Solution Cell Proliferation Assay kit

Cytotoxicity of the film spray on MDCK and Vero cells was also assessed using an MTS-based cytotoxicity assay. After 72 h of incubation, the percentage of viable cells from each concentration of drug treatment was determined for the CC_50_ of the curcumin film spray. Percentages of Vero and MDCK cell viability after treatment with the samples are shown in Fig. 3. The CC_50_ of the film spray at 14.56 and 12.88 µg/ml were found toward Vero and MDCK cells, respectively. Cell viability > 50% was detected at film spray concentrations < 12.5 µg/ml for both cell types. Therefore, the concentration of the film spray at 12.5 µg/ml was used as the maximum concentration for determining the antiviral activity.

### Antiviral activity of the curcumin film spray by plaque reduction assay

**Fig. 4:**
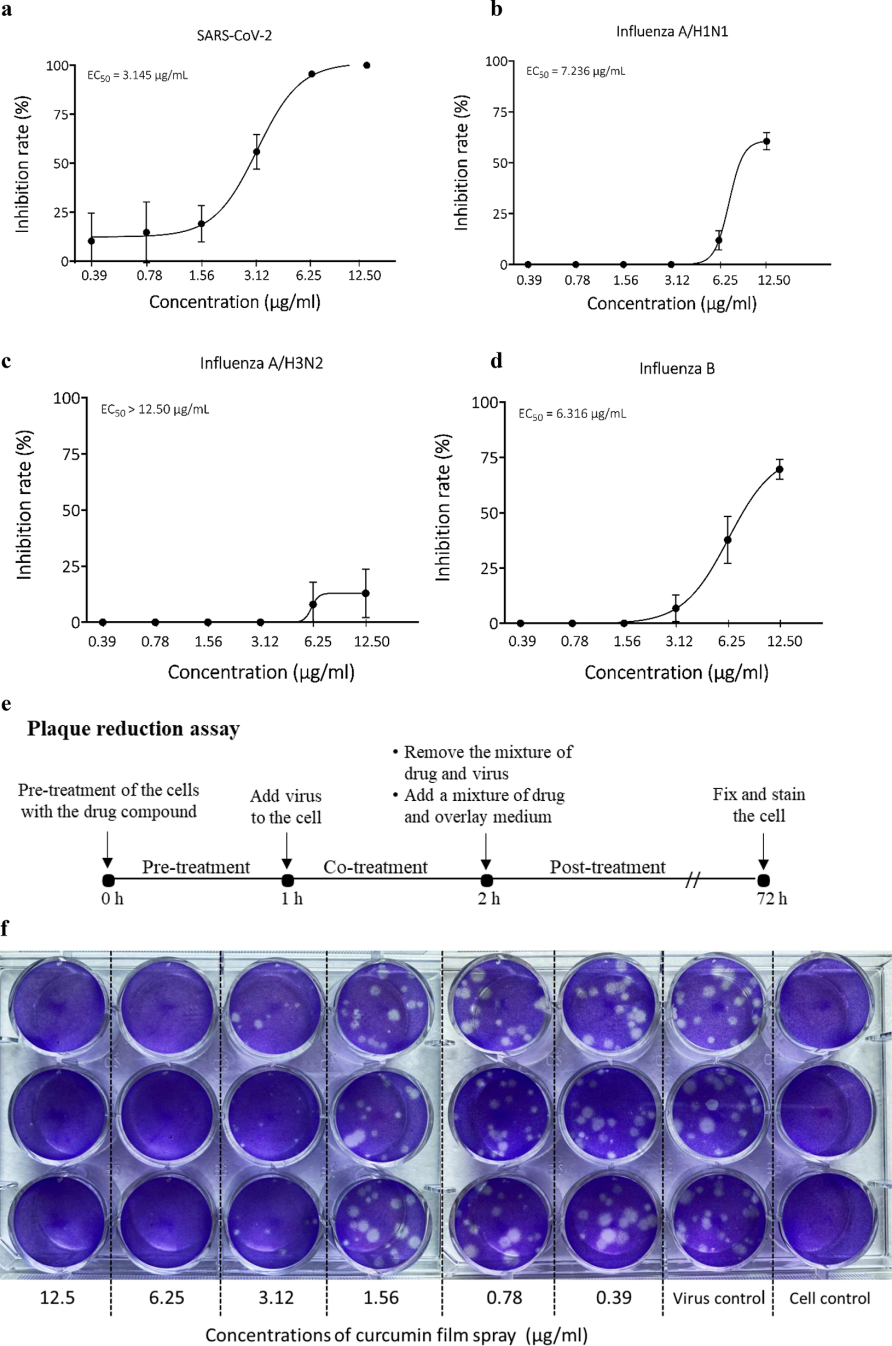
Antiviral effect of curcumin film spray against SARS-CoV-2 examined by plaque reduction assay. Dose-response curve of antiviral activity of curcumin film spray against; (**a**) SARS-CoV-2, (**b**) influenza A/H1N1, (**c**) influenza A/H3N2, and (**d**) influenza B viruses, (**e**) Diagram for antiviral activity screening by plaque reduction assay, (**f**) Photo of plaque reduction assay experiment. Each concentration of the film spray was performed in triplicate wells

The antiviral activity of the film spray against SARS-CoV-2, influenza A/H1N1, A/H3N2, and influenza B was assessed in vitro by plaque reduction assay, and EC_50_ values for each virus were examined. Dose-response curves for the antiviral activities of the film spray samples against all 4 viruses are shown in Fig. 4. The EC_50_ and SI values for each virus are shown in Table 2. Results obtained among the 4 viruses revealed that curcumin film spray demonstrated the highest inhibitory activity against SARS-CoV-2; with the lowest EC_50_ of 3.15 µg/ml (Fig. 4), and highest SI value of 4.62, followed by influenza B (EC_50_ = 6.32 µg/ml, SI = 2.04), and influenza A/H1N1 (EC_50_ = 7.24 µg/ml, SI = 1.78), respectively. On the other hand, it exhibited a slight antiviral effect against influenza A/H3N2 with the EC_50_ > 12.5 µg/ml, SI < 1.03 (Table 1).

**Table 2:** EC_50_ and SI values of curcumin film spray against tested viruses

Test viruses	EC_50_ (µg/ml)	SI (CC_50_/EC_50_ ratio)
SARS-CoV-2 pseudovirus SARS-CoV-2	7.74 3.15	1.68 4.62
Influenza A/H1N1	7.24	1.78
Influenza A/H3N2	> 12.5	< 1.03
Influenza B	6.32	2.04

**Fig. 5:**
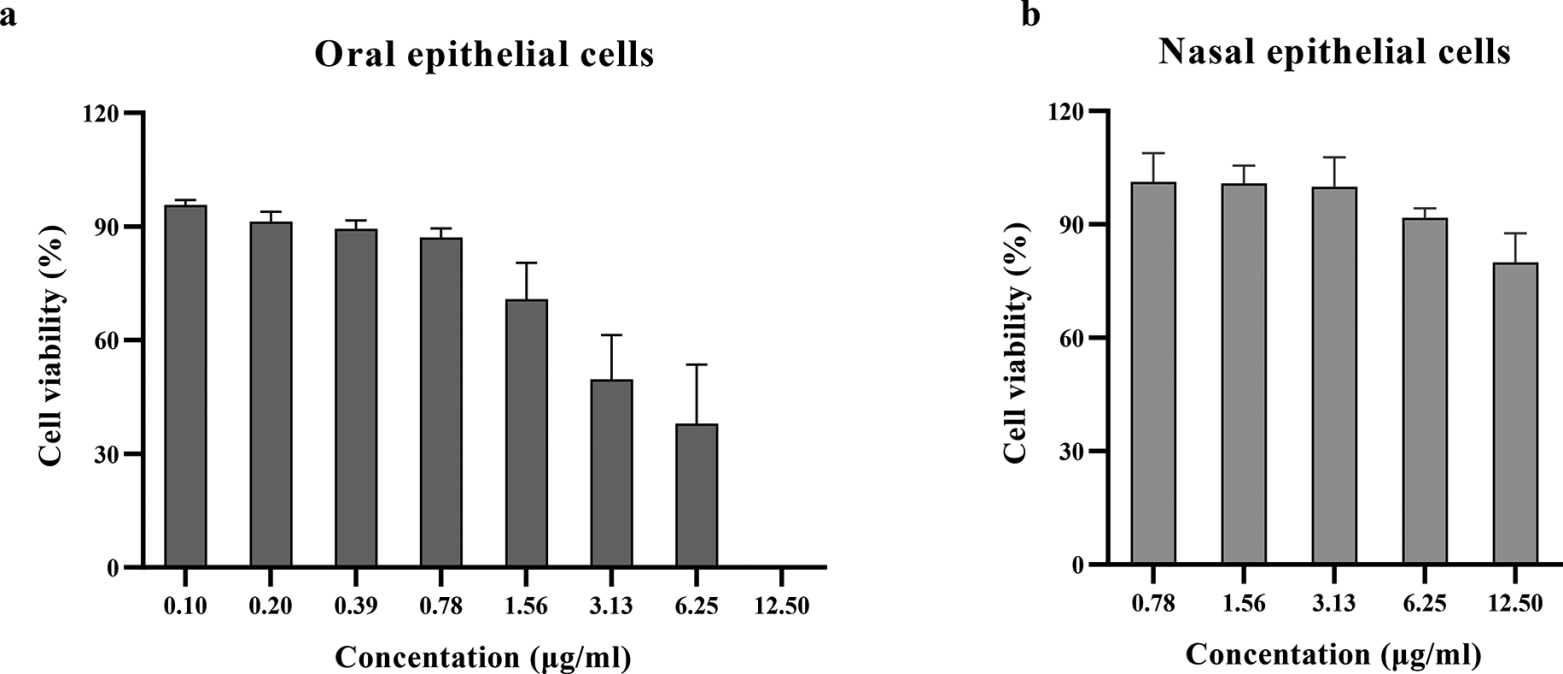
Cytotoxicity of the curcumin film spray on oral keratinocytes (**a**), and nasal epithelial cells (**b**) by MTT assay. The film spray is safe at concentrations of up to 1.56 µg/ml in oral keratinocytes and 12.5 µg/ml in nasal epithelial cells, respectively

The cytotoxicity of the film spray to oral keratinocytes and nasal epithelial cells was also assessed by MTT assay. The results showed that the film spray is safe at the concentration of up to 1.56 µg/ml in oral keratinocytes and 12.5 µg/ml in nasal epithelial cells, respectively (Fig. 5).

### Effects of the film spray on mucosal innate immunity

**Fig. 6:**
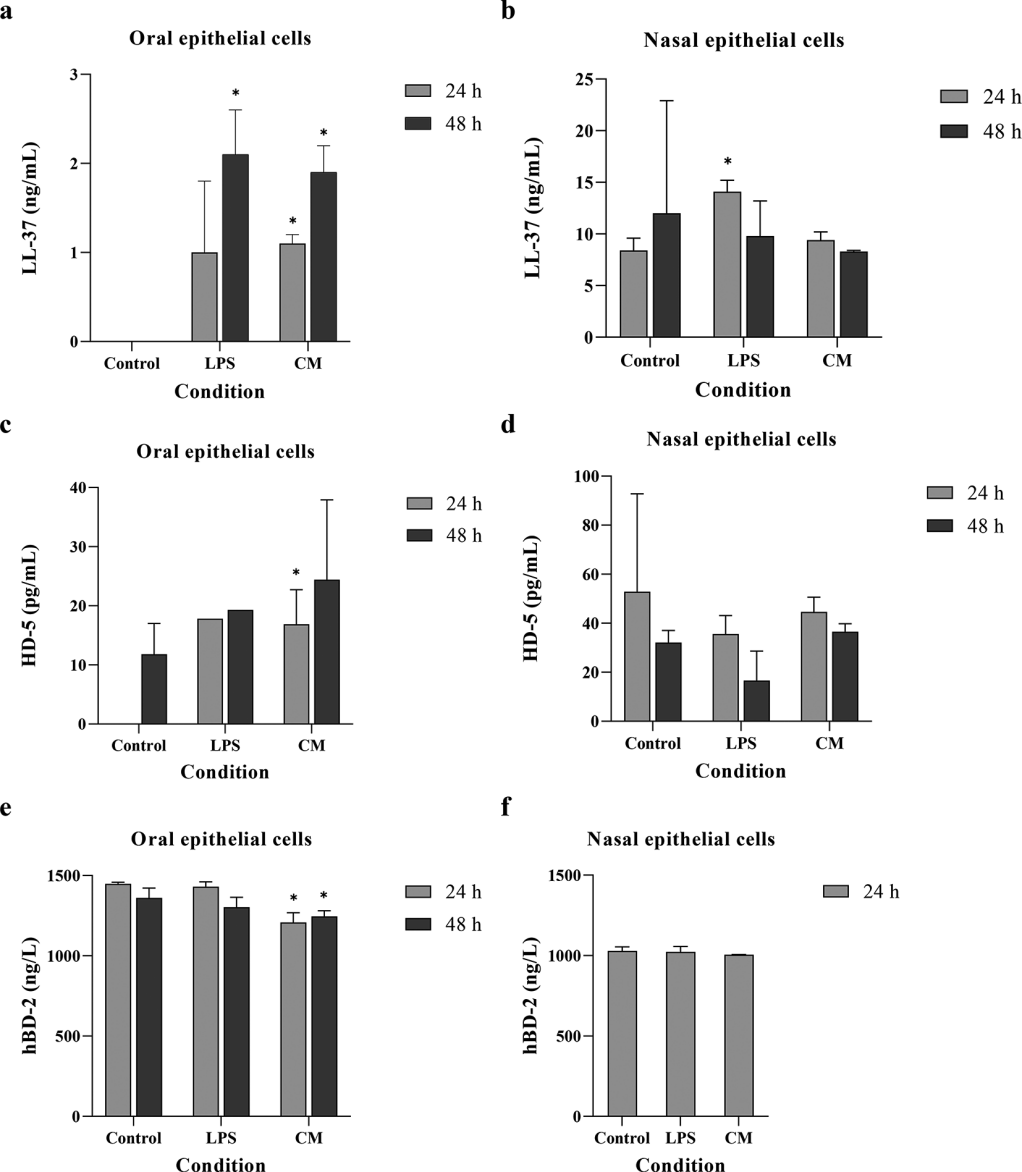
Effects of the curcumin film spray formulation on oral and nasal mucosal innate immunity measured by the level of LL-37 (**a**, **b**), HD-5 (**c**, **d**), and hBD-2 (**e**, **f**) proteins produced by oral keratinocytes and nasal epithelial cells. The spray enhanced the production of LL-37 and HD-5 by oral keratinocytes greater than that of the controls, whereas such effects were not found in nasal epithelial cells. *P*-value was presented as *<0.05 versus untreated control

Oral and nasal innate immune markers, in response to the film spray containing curcumin, were determined by ELISA. The results showed that while the secretion of antimicrobial peptides LL-37 and HD-5 by oral keratinocytes was significantly induced at both 24 and 48 h exposures, no upregulation of LL-37 by nasal epithelial cells was detected at those time points (Fig. 6a–d). The secretion of hBD-2 protein by oral keratinocytes was significantly decreased at both time points, but no changes were detected in nasal epithelial cells (Fig. 6e, f).

### Effects of the film spray on inflammatory cytokines

**Fig. 7:**
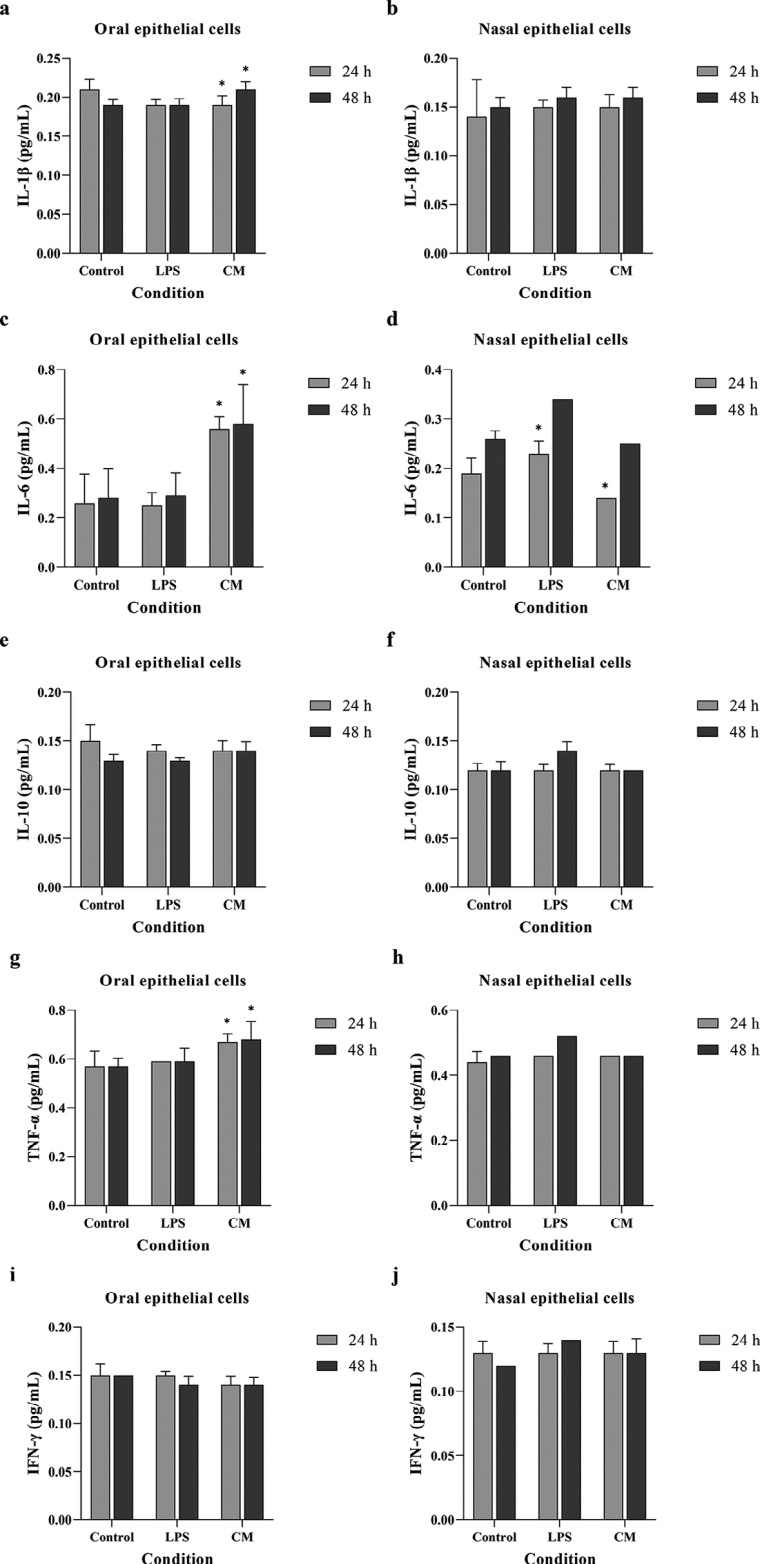
Amount of various cytokines produced by oral keratinocyte and nasal epithelial cells in response to oro-nasal film spray containing curcumin measured by a commercial Milliplex Map Kit; IL-1β (**a**, **b**), IL-6 (**c**, **d**), IL-10 (**e**, **f**), TNF-α (**g**, **h**), IFN-γ (**i**, **j**). The spray enhanced the production of anti-inflammatory cytokine IL-6 and pro-inflammatory cytokine TNF-α produced by oral keratinocytes greater than that of the controls, whereas such effects were not found in nasal epithelial cells. No significant changes in the production of pro-inflammatory cytokines were observed. *P*-value was presented as *<0.05 versus untreated control

Effects of the film spray on inflammatory cytokines were determined by a multiplexed bead immunoassay. The results showed that the film spray containing curcumin upregulated the production of IL-6 and TNF-α by oral keratinocytes, but not nasal epithelial cells. No extraordinary effects on IL-1β, IL10, and IFN-γ were detected (Fig. 7).

## Discussion

**Fig. 8:**
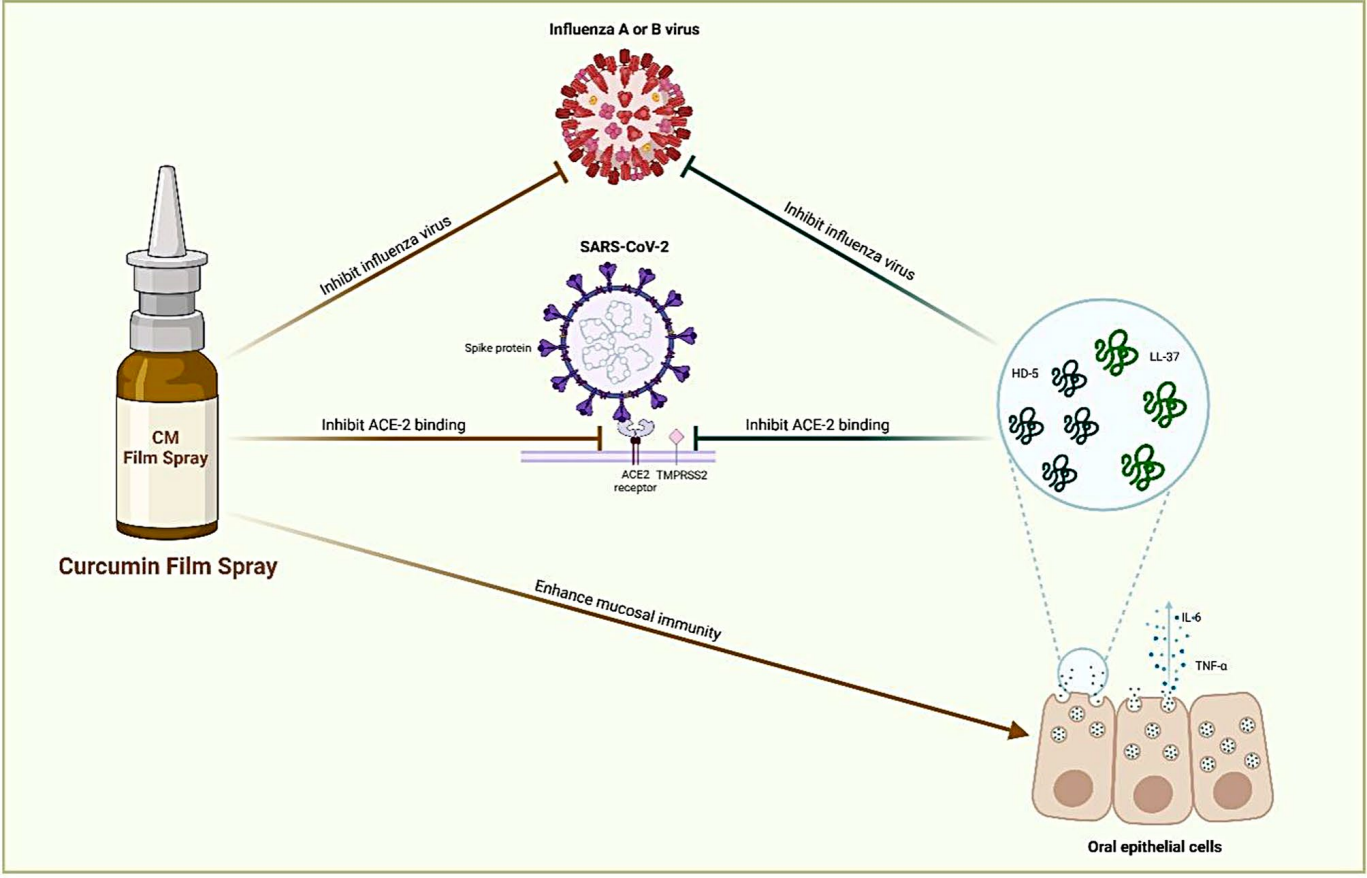
Various actions of the curcumin film spray. Formulation of curcumin film spray shows various actions on prevention of COVID-19; First, it can inhibit SARS-CoV-2 infection by inhibiting ACE-2 binding. Second, the film spray upregulates the production of antimicrobial peptides LL-37 and HD-5 produced by oral epithelial cells, which have been previously reported to inhibit SARS-CoV-2 infection in silico. Third, the film spray induced the production of antiinflammatory cytokines IL-6 and TNF-α produced by oral epithelial cells. In addition, the film spray inhibits influenza virus infection.The infographic was designed by BioRender.com (https://app.biorender.com)

This study demonstrated that curcumin has versatile pharmacological properties including antiviral, immunomodulating, and anti-inflammatory activities. Formulation of the oro-nasal film spray containing curcumin showed potent antiviral activity against both SARS-CoV-2 and influenza virus infection, without cytotoxicity at therapeutic doses. The film spray also modulated mucosal innate immunity by upregulating the production of antimicrobial peptides LL-37 and HD-5, and anti-inflammatory cytokine IL-6 produced by oral keratinocytes. Effects of the curcumin film spray are summarized in Fig. 8.

This study showed that curcumin compound can inhibit SARS-CoV-2 pseudovirus infection in vitro. The film spray containing curcumin can prevent viral infection at the entry step of virus replication with EC_50_ of 7.74 µg/ml. Moreover, antiviral activity from the plaque reduction assay in which curcumin presented throughout steps of viral replication demonstrated that the curcumin inhibited SARS-CoV-2 replication at the EC_50_ of 3.15 µg/ml. According to the inhibition at the entry step by SARS-CoV-2 spike pseudotyped virus infection assay, EC_50_ value was two-fold higher than the plaque reduction assay. These might indicate that curcumin film spray can also inhibit the other steps of in vitro replication. In addition to inhibiting COVID-19, the film spray containing curcumin can inhibit both influenza A and B viruses with similar EC_50_ values of 7.24 and 6.32 µg/ml for A/H1N1 and B viruses, respectively. This study demonstrated that curcumin film spray had stronger antivirus activity against SARS-CoV-2 than influenza viruses. The film spray showed low potential antiviral effect on influenza A/H3N2 virus with an EC_50_ of > 12.5 µg/ml.

The findings of this study revealed that curcumin has pharmacological potential in the prevention and treatment of COVID-19 and flu. However, curcumin’s therapeutic targets and mechanisms of action against the virus have not been clearly established. Previous studies reported that curcumin inhibits the progression of inflammatory diseases, including COVID-19, through different mechanisms [26, 27]. Another study reported that curcumin inhibits influenza infection by interfering with multiple cellular signaling pathways [28]. A study by Dai et al. [29] showed that curcumin can inhibit eight strains of IVA by direct inhibition of viral replication in the adsorption stage. It has been shown that curcumin could suppress adsorption and replication of the virus through Nrf2 pathway to suppress the NF-κB and inflammatory cytokines [29]. Due to its broad pharmacological properties, curcumin has been proposed to replace current influenza therapies [30].

It is recognized that antimicrobial peptides possess broad-spectrum antimicrobial activities along with other biological functions. In humans, there are two kinds of antimicrobial peptides: defensin and cathelicidin family peptides. Defensin family peptide has many members, including HD-5 and hBD2 [31]. Defensins and LL-37 disrupt microbial membranes, leading to pore formation and lysis [17].

LL-37 plays an essential role in protecting humans against infectious diseases by modulating immune cells and regulating the secretion of inflammatory effector molecules [17]. LL-37 has been shown to inhibit SARS-CoV-2 entry via a dual mechanism [19]. A recent study demonstrated that LL-37 can be used along with inactivating HSV-1 as a therapeutic agent against SARS-CoV-2 [32]. Another study found that LL-37 attaches to the SARS-CoV-2 spike protein [33]. LL-37 also shows protective effects during IVA infection [34]. Previous studies demonstrated that LL-37 plays a crucial role in host defense against IVA infection through direct antiviral effects and modulating inflammatory responses to infection [34, 35]. Besides its direct antimicrobial activities against the virus, LL-37 also has important immunomodulatory effects on neutrophil H_2_O_2_ responses to influenza virus [34]. The upregulation of LL-37 by the curcumin film spray may further enhance its action against both SARS-CoV-2 and influenza virus infection.

HD-5, an α-defensin released from Paneth cells in the small intestine, has been reported to block SARS-CoV-2 pseudovirions from entering enterocytes [18]. These may represent innate protection of intestinal cells against viral infection. In the present study, the upregulation of HD-5 by the oral keratinocytes, in response to the film spray, was demonstrated. These properties may further enhance the antiviral activity of the spray against the virus infection.

Human β-defensins (hBDs), produced by epithelial cells either constitutively or in response to inflammatory stimuli, have been shown to inhibit IVA [36–38]. In addition, a study by Zhang et al. [39] demonstrated that hBD-2 inhibited the entry of SARS-CoV-2 into ACE-2-expressing HEK 293T cells in a dose-dependent manner, with an IC_50_ of 2.8 µM. However, in our study, while no changes were observed in the nasal epithelial cell culture, reduced production of hBD-2 by the oral keratinocyte cell culture was noted in response to the film spray. This may indicate that different sites of epithelial cells lining the mucosa differentially express innate immunity.

In the present study, the induction of IL-6 and TNF-α produced by oral epithelial cells in response to the film spray was detected, while no changes were found in nasal epithelial cells. It has been noted that IL-6, as an anti-inflammatory cytokine, plays a vital role in viral infection of the respiratory tract [40]. It has been reported that LL-37 reduces pro-inflammatory cytokine responses during influenza virus infection in vivo [34]. These multi-actions of the film spray make it a promising topical agent in preventing SARS-CoV-2 and influenza virus infection.

Although curcumin has long been used in traditional Asian medicine for its anti-inflammatory and healing properties, its immunomodulatory role remains poorly understood. It has been shown that dietary supplementation with curcumin reduces pro-inflammatory biomarkers while increasing anti-inflammatory mediators [41]. Curcumin has been shown to mediate attenuation of pro-inflammatory IFN-γ, suggesting its role as an immunosuppressant [42]. Our study revealed that the film spray containing curcumin upregulated the production of IL-6 and TNF-α by oral keratinocytes, but not by nasal epithelial cells. No profound effects on pro-inflammatory cytokines IL-1β, IL-10 and IFN-γ were detected. Thus, it is possible that curcumin may act as a double-edged sword on anti-inflammatory and pro-inflammatory cytokines [43]. Alternatively, it is possible that the effects of the film spray may depend on concentration of curcumin, time of exposure and types of the cells.

The pathophysiology of COVID-19 is highly complex, and the mechanisms for how SARS-CoV-2 modulates different systems in the host remain unknown. This heterogenicity may require a compound that has several action potentials. In this regard, a natural compound like curcumin seems to be a promising candidate in the adjuvant treatment of COVID-19, due to its various mechanisms of actions. Resistance to drugs currently used in treatment of influenza virus infection has caused great concern. Consequently, developing new antiviral treatment for this virus is needed. In general, deaths caused by SARS-CoV-2 and IVA infection result from acute lung injury, systemic inflammation, or bacterial superinfection. Searching for compounds with antiviral, antibacterial and anti-inflammation effects would be beneficial. Because of this need, curcumin is a candidate with enormous potential for the treatment of both SARS-CoV-2 and influenza virus infections.

## Conclusion

This study demonstrated that curcumin oro-nasal film spray possesses antiviral activity against SARS-CoV-2 and influenza virus. In addition to antiviral activity, the film spray enhanced the production of LL-37 and HD-5, the two antimicrobial peptides previously reported to inhibit SARS-CoV-2 and IVA infection in silico [19, 34, 35]. These findings indicate that the film spray has potential for preventing both SARS-CoV-2 and influenza virus infections. Furthermore, the film spray modulated the expression of inflammatory cytokine. The infection by SARS-CoV-2 and influenza viruses can induce severe systemic illness through the inflammation induced by the viruses. Thus, the induction of anti-inflammatory cytokines by the film spray emphasizes that it can serve not only as an antiviral but can also dampen inflammatory injury induced by the viruses. These mechanisms of actions by the film spray may be powerful enough to inhibit the viral entry into the host cell, prevent lung injury, and reduce the severity of the disease.

The limitation of this study is that it was performed only in vitro. Further studies should be carried out in an animal model and in clinical trials to confirm the findings. Future studies should also focus on mechanisms involved in inhibition of viral infection by the film spray. Antiviral tests against intact viruses, investigation of mucosal innate immunity, and anti-inflammatory activity in the infected/film spray treated cells should be performed to better understand efficacy of the firm spray. Other antimicrobial peptides produced by oral keratinocytes and nasal epithelial cells in response to the film spray should be further determined. These may lead to finding powerful therapeutic agents against SARS-CoV-2 and influenza virus infection.

## Data Availability

Data and materials are available upon request to the corresponding author, and will be considered on a case-by-case basis.

## Abbreviations

ACE-2: angiotensin-converting enzyme-2
CC_50_: half maximal cytotoxic concentration
CCK-8: Cell Counting Kit-8
COVID-19: coronavirus disease
DMEM: Dulbecco’s Modified Eagle Medium
DMSO: dimethyl sulfoxide
EC_50_: half maximal effective concentration
EMEM: Eagle’s Minimum Essential Medium
FBS: fetal bovine serum
hBD-2: human β defensin 2
HD-5: human defensin 5
HEK: Human embryonic kidney cells
HSV-1: herpes simplex virus type 1
IFNs: interferons
IL: interleukin
IVA: influenza virus type A
LBD: ligand-binding domain
MTT: methylthiazol tetrazolium
NO: Nitric oxide
RBD: receptor-binding domain
OD: Optical density
OKC: oral keratinocyte
SARS-CoV-2: severe acute respiratory syndrome coronavirus-2
TMPRSS-2: Transmembrane protease serine-2
TNF-α: tumor necrosis factor-α
